# Genome-Wide Identification of Calcium-Response Factor (CaRF) Binding Sites Predicts a Role in Regulation of Neuronal Signaling Pathways

**DOI:** 10.1371/journal.pone.0010870

**Published:** 2010-05-27

**Authors:** Andreas R. Pfenning, Tae-Kyung Kim, James M. Spotts, Martin Hemberg, Dan Su, Anne E. West

**Affiliations:** 1 Program in Computational Biology and Bioinformatics, Duke University Medical Center, Durham, North Carolina, United States of America; 2 Department of Neurobiology, Harvard Medical School, Boston, Massachusetts, United States of America; 3 F.M. Kirby Neurobiology Center and Departments of Neurology and Neurobiology, The Children's Hospital Boston, Boston, Massachusetts, United States of America; 4 Department of Ophthalmology, The Children's Hospital Boston, Boston, Massachusetts, United States of America; 5 Department of Neurobiology, Duke University Medical Center, Durham, North Carolina, United States of America; Yale School of Medicine, United States of America

## Abstract

Calcium-Response Factor (CaRF) was first identified as a transcription factor based on its affinity for a neuronal-selective calcium-response element (CaRE1) in the gene encoding Brain-Derived Neurotrophic Factor (BDNF). However, because CaRF shares no homology with other transcription factors, its properties and gene targets have remained unknown. Here we show that the DNA binding domain of CaRF has been highly conserved across evolution and that CaRF binds DNA directly in a sequence-specific manner in the absence of other eukaryotic cofactors. Using a binding site selection screen we identify a high-affinity consensus CaRF response element (cCaRE) that shares significant homology with the CaRE1 element of *Bdnf*. In a genome-wide chromatin immunoprecipitation analysis (ChIP-Seq), we identified 176 sites of CaRF-specific binding (peaks) in neuronal genomic DNA. 128 of these peaks are within 10kB of an annotated gene, and 60 are within 1kB of an annotated transcriptional start site. At least 138 of the CaRF peaks contain a common 10-bp motif with strong statistical similarity to the cCaRE, and we provide evidence predicting that CaRF can bind independently to at least 64.5% of these motifs *in vitro*. Analysis of this set of putative CaRF targets suggests the enrichment of genes that regulate intracellular signaling cascades. Finally we demonstrate that expression of a subset of these target genes is altered in the cortex of *Carf* knockout (KO) mice. Together these data strongly support the characterization of CaRF as a unique transcription factor and provide the first insight into the program of CaRF-regulated transcription in neurons.

## Introduction

The function of any given transcription factor is determined in large part by its DNA binding specificity, which defines its potential target genes. Over 1000 gene products are annotated as transcription factors in the mammalian genome, the vast majority of which belong to large families classified by the homology of their DNA binding domains (e.g., homeodomain, zinc finger, bHLH) [1]. Individual transcription factors within a family often serve related functions [2] and may compensate at least in part for the loss of other family members [3]. This redundancy may have supported the diversification of transcriptional mechanisms during evolution and the development of increasing organismal complexity [4].

Some of the most important recent insights into transcription factor biology have come from the application of technologies that capture the full complement of transcription factor binding sites across the genome [5]. By using chromatin immunoprecipitation followed either by hybridization to tiled genomic microarrays (ChIP-chip) or high-throughput sequencing (ChIP-Seq) it is possible to identify a large, unbiased set of transcription factor binding sites, suggesting candidate target genes [6], [7]. Studies of this kind have been used to reveal unexpected sequence variation between the individual binding sites selected by a single transcription factor. Furthermore by elucidating large sets of potential target genes, these data may suggest new functions for a transcription factor in previously unanticipated cellular processes [6], [7], [8].

Transcription factors are essential for accommodating intracellular states to extracellular stimuli. In the nervous system, transcription factors play an important role in coordinating neuronal responses following changes in synaptic activity - a key stimulus for shaping brain development, driving synaptic plasticity, and promoting survival of mature neurons [9]. The importance of this process is highlighted by the fact that mutations in a large number of transcription factors and transcriptional co-regulators, including *MECP2*, *FOXP2*, *CBP*, and *GTF2I/GTF2IRD1*, are associated with cognitive impairment in humans [10]. Activity-regulated transcription of the gene encoding the neurotrophin BDNF is essential for the function of this gene product both in synaptic plasticity and brain development [11], [12], thus substantial effort has been devoted to understanding the specific mechanisms that regulate transcription of this gene [13].

Calcium-Response Factor (CaRF) is a novel nuclear protein first identified as a binding protein for a calcium-response element (CaRE1) in *Bdnf* promoter IV [14]. In overexpression assays CaRF acts as a CaRE1-dependent transcriptional activator, however other than *Bdnf* none of CaRF's target genes are known. Surprisingly, CaRF shares no sequence similarity with any known family of transcription factors, and it has no homologs in the mammalian genome. Given this evidence that CaRF is a unique transcription factor, we set out to characterize CaRF's DNA binding properties and identify its potential target genes that might in turn suggest biological functions for CaRF beyond *Bdnf* regulation.

Here we show that the DNA binding domain of CaRF has been highly conserved over evolution, and we use both *in vitro* binding site selection and genome-scale *in vivo* chromatin immunoprecipitation followed by sequencing (ChIP-Seq) as tools to identify and characterize high affinity CaRF-binding DNA sequences. These data reveal a large set of putative CaRF target genes in mouse cortical neurons, providing the first insight into the CaRF regulon.

## Materials and Methods

### Animals

*Carf* exon 8 KO mice were generated by homologous recombination [15]. All animal procedures were approved by the Duke University Institutional Animal Care and Use Committee. Veterinary care was provided by the staff of the Duke University Department of Laboratory Animal Research, an AAALAC accredited facility. Animals were euthanized following procedures that are in accordance with the recommendations of the Panel on Euthanasia of the American Veterinary Medical Association.

### Bacterial synthesis of CaRF protein

Full-length mouse CaRF was subcloned in the bacterial expression vector pThioHisA (Invitrogen, Gaithersburg, MD) at the *EcoRI* and *XbaI* sites. The construct was transformed into Top10 *E. coli* and expression of CaRF was induced for 3 hours with 0.1mM IPTG. Bacteria were lysed by sonication and CaRF was purified over a ProBond nickel resin column. After washing to remove unbound protein, binding was competed with 50mM imidazole to remove nonspecific proteins and then CaRF was eluted with 250mM imidazole. Fractions containing CaRF as determined by Western analysis were pooled and concentrated to a final concentration of 100ng/uL.

### Electrophoretic Mobility Shift Assay (EMSA)

EMSAs were performed as described [14] using *in vitro* transcribed and translated (TNT) protein (Promega, Madison, WI) or bacterially expressed CaRF as described above. 2µL TNT or purified CaRF protein was incubated with 50fmol ^32^P nick labeled (Amersham/GE Healthsciences, Piscataway, NJ) or polynucleotide kinase (New England Biolabs; Ipswitch, MA) end-labeled annealed oligonucleotide probes prior to separation on 6% non-denaturing acrylamide gels. In competition assays, unlabeled probes were added to nuclear extracts in 100-fold molar excess (unless otherwise noted) to the TNT protein for 30 min. prior to addition of the radiolabeled probe. Gels were dried and visualized by phosphorimager (GE Healthsciences). Mobility-retarded bands were quantified relative to probe intensity in the same lane using the ImageQuant image analysis program (Molecular Devices, Sunnyvale, CA). Oligonucleotide probes are listed in **Table S1**.

### Binding site selection screen

PCR-assisted binding site selection was performed essentially as described [16]. Oligonucleotide sequences and PCR primers used for the screen are listed in **Table S1**. Full length human CaRF (hCaRF) was tagged at its N-terminus with the FLAG-epitope (GACTACAAGGACGATGACGATAAA) and used for in vitro TNT as above. For the binding selection, random 16mer nucleotide sequences were synthesized (IDT DNA, Coralville, IA) within a 66bp oligo, then made double-stranded by a single round of PCR using primers against the flanking sequences. Oligos were incubated with TNT hCaRF or a control TNT master mix and immunoprecipitated with the M2 anti-FLAG epitope antibody (Sigma, St. Louis, MO). Coimmunoprecipitated oligos were purified and amplified by PCR, then used for an additional three rounds of selection. A subset of the final samples were radiolabeled and tested by EMSA for their ability to bind hCaRF. The remaining samples were ligated into pBSK (Stratagene, La Jolla, CA), the inserts were sequenced and 62 were aligned using ClustalW (http://www.clustal.org/) [17] to reveal the consensus CaRF binding motif. The cCaRE logo was generated using the WebLogo program (http://weblogo.berkeley.edu/) [18].

### Chromatin Immunoprecipitation

Chromatin Immunoprecipitation (ChIP) was performed as described [19]. For CaRF ChIP-Seq, separate cultures of cortical neurons from newborn *Carf* ^+/+^ (WT) or *Carf* ^−/−^ (KO) pups were plated at a density of 10 million cells/10cm dish. 50 million neurons of each genotype were used for ChIP. Because the *Carf* mice are on a mixed C57BL6/129SvJ background, WT and KO siblings were crossed (WTxWT and KOxKO) to generate the pups for this experiment in order to minimize genetic background variations. At 4DIV, cultured neurons were treated overnight with tetrodotoxin (TTX, 1µM; Calbiochem, La Jolla, CA), then on DIV5 protein-DNA complexes were processed for ChIP. 4µL purified anti-CaRF antibody (#4510)[15] was added to each lysate (WT and KO) and incubated overnight with rotation at 4°C. For ChIP on the *Carf* promoter, striatum was dissected from brains of *Carf* WT or KO mice and snap frozen in liquid nitrogen. 3–5 independent samples were pooled, homogenized, and processed for ChIP as above. Immunoprecipitations were performed at 4°C overnight with 5–10µg of each specific antibody and assayed by real-time PCR using SYBR green detection. Antibodies used in this study include purified normal mouse polyclonal IgG (Millipore, Billerica, CA cat. #12-371) and anti-RNA polymerase II, clone CTD4H8 which recognizes the largest subunit of RNA polymerase II (Millipore, cat. #05-623). For input samples, 25µl (6–7% of the amount used for IP with specific antibody) was added to 75µl of elution buffer, NaCl was added to 200mM final concentration, and samples were reverse crosslinked at 65°C overnight. All ChIP pulldowns are displayed as % of input for each sample (WT or KO). Data presented are the result of two independent experiments, and statistical significance was evaluated by a Student's two-tailed unpaired t-test.

### ChIP library construction and sequencing

DNA fragments coimmunoprecipitated with CaRF were repaired using the End-It DNA End Repair Kit (Epicentre Biotechnologies, Madison, WI), purified using the MinElute Reaction Cleanup Kit (Qiagen, Valencia, CA) and eluted in 20µl EB buffer. The resulting DNA fragments were ligated to adaptors for the SOLiD sequencer (Life Technologies, Carlsbad, CA) for 20 minutes at RT using the Quick Ligase Kit (NEB), followed by purification using the MinElute Reaction Cleanup Kit. Sequences of adaptor and barcodes can be found at http://www.appliedbiosystems.com. The purified adaptor-ligated ChIP DNA fragments were subject to 6% native-PAGE for In-Gel PCR reaction. A gel slice containing 175∼200bp adaptor-ligated ChIP DNA fragments was cut and shredded. Then to add bar codes, PCR Platinum Supermix (100∼200µL, Invitrogen), 50pmol of PCR primers containing bar codes, 0.5µl Taq DNA polymerase (NEB), and 0.15µl *Pfu* Turbo DNA polymerase (Stratagene) were added into the shredded gel slice. The adaptor-ligated ChIP DNA fragments were amplified by 15 cycles of In-Gel PCR reactions. After the PCR reaction, gel pieces were filtered out by 0.45µm filter spin column and the amplified ChIP-Seq library was purified by MinElute PCR purification kit (Qiagen). The library was purified by one more round of 6% PAGE. A gel slice containing 200∼250bp PCR products was cut and shredded, and the amplified library was extracted out of the gel by passive elution in elution buffer (1.5M ammonium acetate in 1× TE). Gel pieces were filtered out by filter spin column and both ChIP-Seq libraries were purified by Qiaquick PCR purification kit (Qiagen). Samples were affixed to a slide and sequenced on a SOLiD sequencer version 2. After filtering with 3bp mismatches allowed in 35bp reads, just over 5 million uniquely mapped reads (about 40% of total reads) were obtained for each library (WT and KO). The resulting sequences were formatted for alignment in the UCSC genome browser.

### CaRF Wildtype ChIP Peak Finding

To determine CaRF-specific binding sites, we developed an algorithm to identify the sequences specifically enriched in the ChIP samples from *Carf* WT neurons relative to the negative control ChIP samples from *Carf* KO neurons. First we determined the false detection ratio by using a sliding window with a width of 240bp for every 10bp in the mouse genome. Repetitive regions are excluded from our analysis since it is impossible to assign 35bp reads uniquely to such regions [20], [21]. For each window, we calculated the statistic D = R−N where R is the number of reads in the WT sample, and N is the number of reads from the negative control KO sample. By considering the marginal distributions of R and N, we note that they both can be well approximated by a Poisson distribution with parameters λ_R_ and λ_N_, respectively. It follows that D is a Skellam distribution [22]

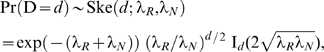
(1)where *I_d_* is the modified Bessel function of the first kind of order *d*. The construction of the null distribution from Eq. (1) takes unequal numbers of reads in the two samples into account by shifting the mode of the distribution. To determine the number of reads required for a 240bp window to be significant, we use the local false detection rate (locFDR) framework [23]. Using this methodology, we assume that the density of *D*, *f*(*D*), can be written as the mixture *f*(*D*) = *p_0_f_0_*(*D*)+*p_1_f_1_*(*D*), where *f_0_* is the null density, *f_1_* is the density of windows corresponding to true peaks and *p_0_*+*p_1_* = 1 with p_0_ 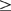
0.9. The locFDR, fdr(*d*) is related to the more familiar FDR [24] through
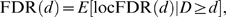
(2)where *E* is the expectation with respect to the mixture density *f*. For a fixed threshold *f*_p_ = 0.01 and empirically estimated λ*_R_* = 0.15273 and λ*_N_* = 0.21607, we find that the critical difference is *d*_0_ = 6 fragments. Inserting the empirical distribution and the Skellam null distribution from Eq. (1), we find that the FDR is ∼1×10^−7^.

### Motif Discovery

Regions of the genome determined to be specifically bound by CaRF were downloaded using the GALAXY [25] platform based on genome coordinates. Due to noise in the exact positioning of the sequence, when significantly bound sequences were found to be within 100bp of each other, the coordinates were expanded to produce one larger region. In addition, very short identified regions (⇐20bp) were discarded from the analysis because these are too short to meaningfully search for 10–15bp motifs. The remaining 144 sequences had a mean length of 112bp, a median of 100bp, and standard deviation of 79bp. These sequences were fed into the PRIORITY motif finder, which uses a Gibbs sampling strategy [26], [27]. A uniform prior was used to search across CaRF bound regions on the positive and negative strand. The default parameters of 20 trials and 10,000 iterations per trial were used. A third order background model was used, although the results were robust to the use of both first and second order. Varying motif lengths were attempted to identify the correct consensus length. At motif lengths lower than 10bp, only a part of each motif resembled the CaRF consensus site. At motif lengths larger than 10bp, the information content was low at peripheral base pairs. Consistently, a significant motif was identified with score 131. As a control, a motif discovery was performed on the same number of sequences with the same length, but from nearby genomic regions. The maximum score of an identified motif in this data set over many trials was only 15, suggesting that the motif discovered in the CaRF ChIP-Seq data is highly significant.

### Quantitative PCR

For evaluation of CaRF target gene expression, brains from single P0 pups from a *Carf* heterozygote (HET×HET) cross were removed, the cortex was dissected and rapidly frozen on liquid nitrogen, then the thawed tissue was used for RNA harvesting. For evaluation of *Carf* mRNA expression, brains from single P0 pups from a *Carf* HET×HET cross were used for neuron culture as described above. Tail biopsies were clipped during dissection and genotyped prior to sample harvesting to identify WT and KO pups. On day 5 in culture, RNA was harvested, and cDNA was synthesized as above. All primers used in this analysis are listed in **Table S1**. To measure *Carf* mRNA we used primers against exons 11–12, distal to the *Carf* exon 8 deletion. Samples were normalized to *Gapdh* as a control for sample handling. Data shown for *Carf* are the result of measurements in 8 individual pups of each genotype. Data for other *Carf* target genes are the result of measurements in 4–6 individual pups of each genotype. For quantitation of chromatin immunoprecipitation of RNA polymerase II on the *Carf* gene, the primers listed in **Table S1** were used to amplify a 173bp region flanking the ChIP peaks in *Carf* exon 1. Statistical significance was determined by a Student's unpaired 2-tailed t-test, and *p*<0.05 was considered significant.

## Results

### CaRF is an evolutionarily conserved, sequence-specific DNA binding protein

**Figure 1:**
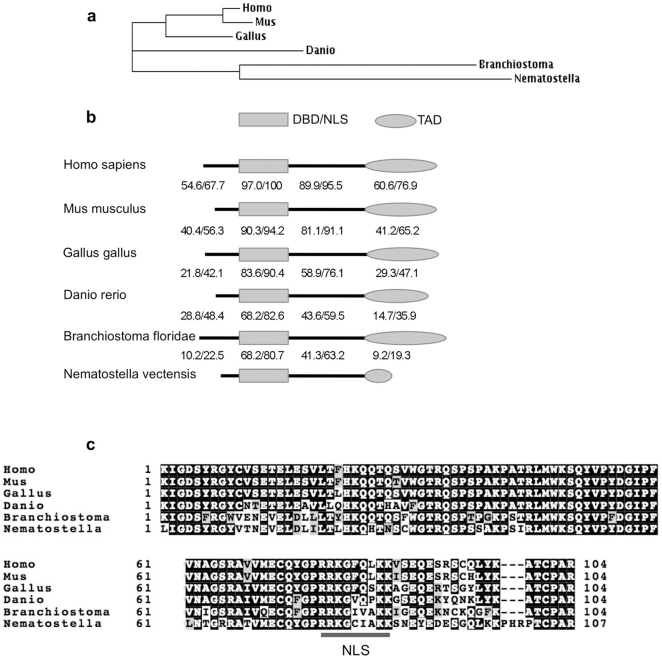
The CaRF DNA binding domain is highly conserved across evolution. CaRF amino acid sequences were obtained from the NCBI and Ensembl databases by BLAST similarity to mammalian CaRF (**Table S2**). Sequences were aligned using ClustalW. a) Phylogram representing the evolutionary distances between CaRF sequences in six species. b) Percent identity and similarity among amino acids in each domain of CaRF. The diagrams are drawn to scale and show four distinct domains of CaRF [14]. From left to right these are the N-terminus (corresponding to human coding exons 1–5), the DNA binding domain and nuclear localization signal (DBD/NLS, coding exons 6–7), an intermediate domain (coding exons 8–10), and the transcriptional activation domain (TAD, coding exons 11–14). The numbers between each pair of sequences show the percent of amino acids within that domain that are identical/conserved between that pair within each domain. Identity and conservation of amino acids were called by ClustalW, and insertions were scored as non-conserved amino acids. c) Sequence alignment of the DBD/NLS domain across all six species. Identical amino acids are highlighted black, conserved amino acids are gray and nonconserved changes are white.

Showing that a protein domain is conserved over evolution is one way of suggesting its importance for function of the protein. Sequence analysis of CaRF against build 37 of the *Mus musculus* genome reveals no significant homology of this protein to any other gene product. However we find gene products with significant similarity to CaRF at the amino acid level in 37 different species (**Table S2**), including 35 vertebrates and the deuterostome *Branchiostoma floridae* [28]. In all species only one gene product with similarity to CaRF can be identified. Interestingly, despite the absence of any gene products with significant similarity to CaRF in the *Drosophila melanogaster* or *Caenorhabitas elegans* genomes, a conserved CaRF ortholog is present in the genome of the cnidarian starlet sea anemone *Nematostella vectensis*, which diverged from the vertebrate lineage over 700 million years ago [29] (**Figure 1a**). Sequence conservation of CaRF orthologs is greatest within the domain required for DNA binding [14], in which 59% of the amino acids are identical across species (**Figure 1b,c**). A second highly conserved region aligns with amino acids 292–472 of mouse CaRF (**Figure 1b**), although the functions of this domain are not known.

**Figure 2:**
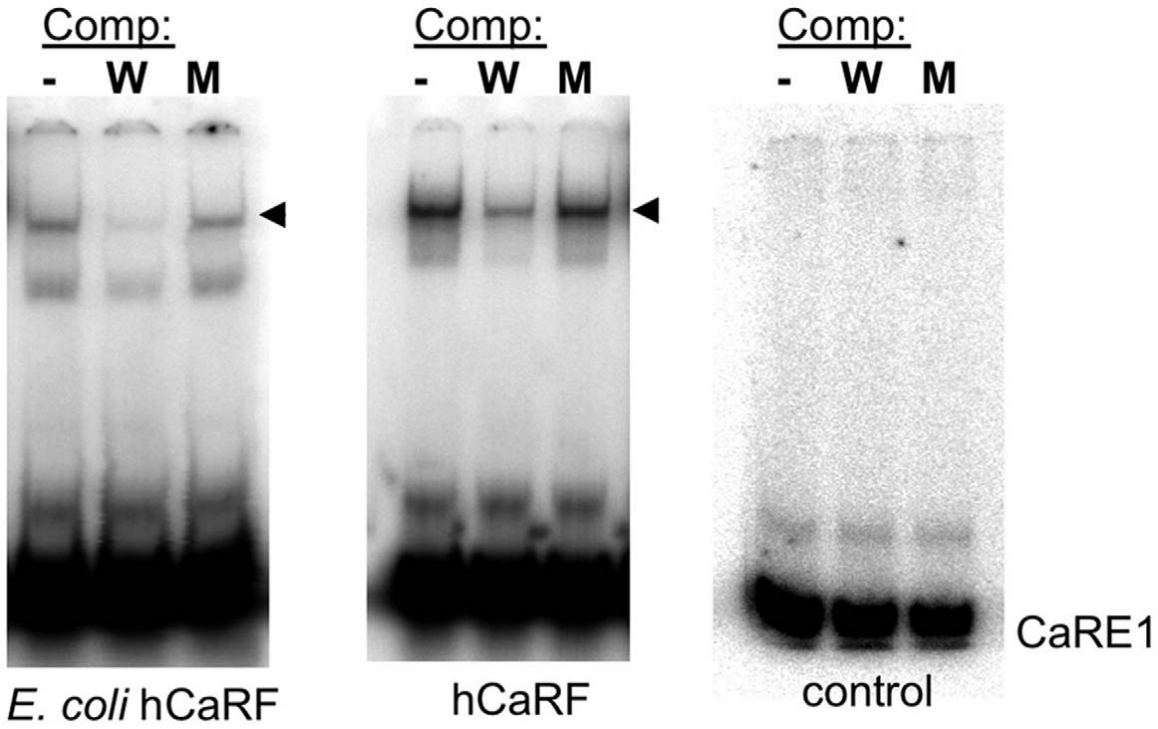
CaRF binds DNA directly. Human CaRF was expressed in bacteria (E. Coli hCaRF) or synthesized in vitro by TNT (hCaRF). Rabbit reticulocyte lysate without CaRF expression was used as control. 2µL of CaRF protein or TNT control was incubated with radiolabeled CaRE1 oligos in the absence (-) or presence of a 50-fold molar excess of competing unlabeled wildtype (W) or mutant (M) CaRE1 probe. Unbound probe is at the bottom of the gel. Arrowhead indicates the complex between CaRF and CaRE1.

Because CaRF does not belong to a previously known DNA binding domain protein family, we wanted to verify that CaRF is capable of binding directly to DNA in the absence of other eukaryotic cofactors. We find that His-tagged CaRF purified from *E. coli* binds *Bdnf* CaRE1 by EMSA and is competed with the same specificity as FLAG-tagged CaRF synthesized using a eukaryotic in vitro transcription and translation system (**Figure 2**). Specifically, the binding of CaRF to CaRE1 can be competed by addition of an excess of unlabeled wildtype CaRE1 probe, but not by the addition of a mutant CaRE1 sequence (mCaRE) that does not support calcium-dependent *Bdnf* transcription [14]. These data establish that CaRF is a conserved, sequence-specific, direct DNA binding protein, strongly supporting its proposed role as a transcriptional regulator.

### Characterization of a high-affinity CaRF binding motif

**Figure 3:**
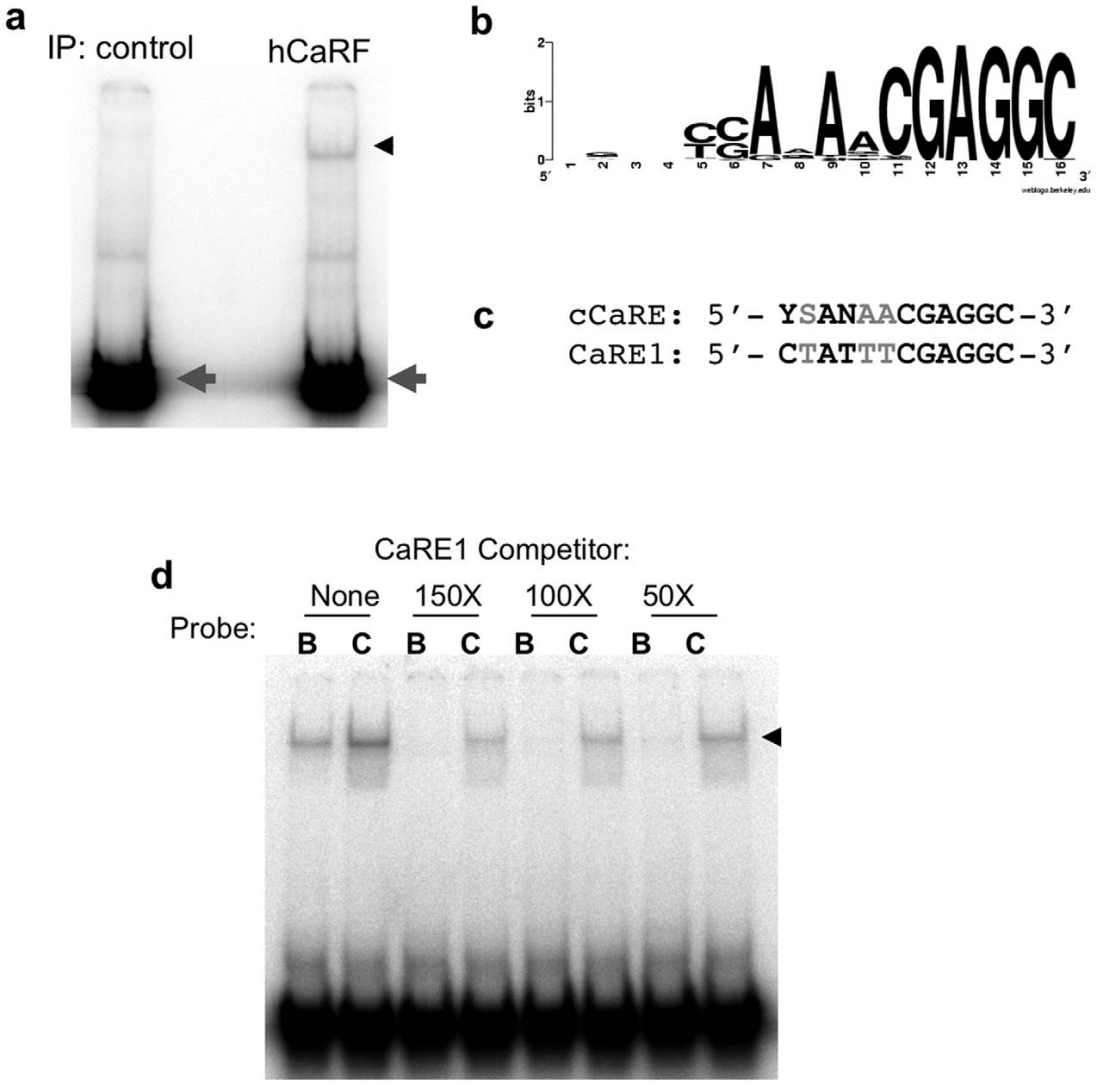
Identification of a consensus CaRF binding element. hCaRF synthesized by TNT (hCaRF) or control rabbit reticulocyte without CaRF (control) was used to coprecipitate oligonucleotides from a library of random 16mers. a) After four rounds of enrichment and amplification, the final pulldown from each sample was radiolabeled and mixed with hCaRF for evaluation by EMSA. Equal amounts of radiolabeled oligos are present in each pool (gray arrowhead), however a CaRF binding band is retarded only from the pool that was isolated by coprecipitation with hCaRF (black arrowhead). b) WebLogo (http://weblogo.berkeley.edu/) representation of the cCaRE consensus motif derived from the 62 sequences in **Table S3**. The position of the bases is indicated along the bottom from 1–16, and the height of the letters indicates the enrichment of that base at each position. If all four bases were equally likely to be present at any position, no base is indicated. c) Alignment of the cCaRE and CaRE1 motifs. Black indicates bases that are conserved between the elements, and gray shows bases that vary. Y = C/T, S = C/G, and N = any base. d) Comparison of the affinity of CaRF for CaRE1 and cCaRE. A constant amount of hCaRF was bound to radiolabeled CaRE1 (B) or cCaRE (C) probes and the relative affinity of the interactions were assessed by competition EMSA upon the addition of a 150, 100, or 50-fold molar excess of unlabeled CaRE1 probe. The band retarded upon CaRF binding is indicated by the arrowhead.

Although we demonstrated previously that CaRF can bind the CaRE1 element of *Bdnf* promoter IV [14], the full range of sequences that can be bound by CaRF was not known. To identify DNA sequences with high affinity for CaRF, we performed a PCR-assisted binding site selection screen. Following four rounds of binding site selection, EMSA analysis of the oligonucleotide pool coimmunoprecipitated with CaRF reveals a strong CaRF-binding band, whereas there is no significant binding to CaRF in a control oligonucleotide pool (**Figure 3a**). The oligos that coimmunoprecipitated with CaRF were cloned and sequenced, then 62 sequences (**Table S3**) were aligned, revealing a 12bp consensus CaRF binding motif (cCaRE) with the sequence 5′- YSANAACGAGGC - 3′ (Y = C/T, S = C/G, and N = any base; **Figure 3b**). This sequence shares significant similarity with the CaRE1 element from the *Bdnf* gene (**Figure 3c**) strongly supporting our previous identification of CaRF as a CaRE1 binding protein. However in competition EMSAs, CaRF shows higher affinity for the cCaRE compared with CaRE1 (**Figure 3d**). Because the cCaRE motif aligns in many of our oligos with a sequence that was used to flank one side of the random 16mer sequence, it is possible that our selection does not fully represent the sequence variability that may be tolerated by CaRF. Nonetheless, these studies indicate that the cCaRE defines a new high affinity CaRF consensus binding site.

### ChIP-Seq identifies genomic binding sites of CaRF in neurons

Given this evidence that CaRF is a direct sequence-specific DNA binding protein, we wanted to find endogenous gene targets of CaRF. To identify CaRF binding sites genome-wide, we performed chromatin immunoprecipitation with an anti-CaRF antiserum from cultured *Carf* WT postnatal mouse cortical neurons followed by sequencing of the coimmunoprecipitated genomic DNA fragments (ChIP-Seq). Mapping these fragments to the reference genome gives a profile of the DNA regions that are enriched in the pulldown, however the challenge is to determine which of the enriched regions (“peaks”) are statistically significant at a given threshold and which correspond to the genomic background. Thus a peak is defined as region that contains significantly more reads from an experimental pulldown than from a negative control. In this case, for our negative control we performed ChIP with the same anti-CaRF antiserum from neuronal cultures made from *Carf* KO mice, which are null for CaRF protein. Independent libraries were constructed from the genomic DNA coimmunoprecipitated from the WT and KO cells, and genomic regions specifically bound by CaRF (CaRF ChIP peaks) were identified using the statistical algorithm described in detail in the Methods section.

**Figure 4:**
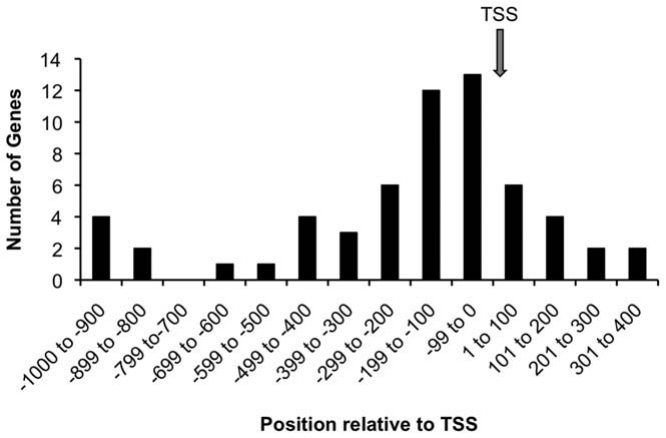
CaRF ChIP-Seq peaks are enriched near transcription start sites. The ChIP peaks from **Table S4** were viewed in the UCSC genome browser (http://genome.ucsc.org) and the distance from the center of each peak to the nearest annotated transcription start site (TSS) was calculated. For the 60 peaks within 1kB of a TSS we tallied the number within each 100bp. The arrow shows the position of the TSS.

176 CaRF ChIP peaks ranging in size from 10–630bp were identified genome-wide (**Table S4**). 128 of the 176 peaks (73%) are within 10kB of an annotated gene, and 60/128 (47%) of these are within 1kB of the annotated transcriptional start site (TSS). Graphing the position of each of these peaks relative to the TSS shows an enrichment in the 200bp just 5′ to the TSS (**Figure 4**). This positional information demonstrates a highly significant enrichment of proximal gene promoters in the CaRF ChIP peaks relative to their overall representation in the genome, raising the possibility that CaRF may contribute to transcriptional regulation of nearby genes.

**Figure 5:**
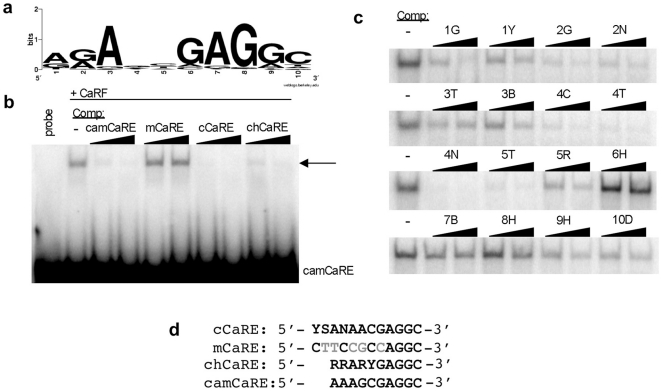
Identification and characterization of a conserved CaRF-binding motif in the CaRF ChIP-Seq peaks. a) WebLogo (http://weblogo.berkeley.edu/) representation of the 10bp motif discovered by the PRIORITY motif finder in the ChIP peak sequences. The height of each letter represents the enrichment of that base at each position. If all four bases are equally represented, no base is shown at that position. b) Competition EMSA analysis of CaRF binding to the consensus chCaRE motif in the ChIP peak of the *Camk2n1* gene (camCaRE). Arrow indicates the CaRF-camCaRE complex, and the right triangles indicate increasing concentrations (50 or 100 fold molar excess) of the unlabeled competitor probes. c) Competition EMSA analysis to examine the relative importance of each base across the 10bp chCaRE motif. Recombinant CaRF was incubated with radiolabeled camCaRE in the absence (-) or presence of a 50 or 100-fold molar excess of unlabeled competitor probes. The right triangle indicates increasing competitor concentrations. Competitor probes were based on the camCaRE sequence (AAAGCGAGGC) with the indicated changes at each position (e.g. 1G has a G rather than an A at position 1 of the motif while the rest of the motif is unchanged). Degenerate code: Y = C/T, N = A,C,G, or T, B = C,G, or T, R = A/G, H = A, C, or T, D = A, G, or T. d) Alignment of the cCaRE, mCaRE, chCaRE, and camCaRE sequences. The mCaRE, which fails to bind CaRF, differs from the CaRF binding sequences at 5 positions, which are shown in gray. Degenerate bases are as described above along with S = C/G.

To determine if the ChIP peaks contain a CaRF binding motif, we extracted the sequences of the 176 CaRF ChIP peaks and subjected them to analysis using the PRIORITY motif finder, which can be used to detect statistically significant common short sequences within a set [26], [27]. 32 ChIP peaks smaller than 20bp were eliminated from the analysis because they are too short to be searched. This analysis found a common 10bp sequence motif in 138 of the 144 sequences examined (**Table S4**). The motif extracted from this set of sequences (chCaRE) has the sequence 5′-RRARYGAGGC-3′, (R = A/G, and Y = C/T) (**Figure 5a**). This sequence is strikingly similar to the high affinity cCaRE (5′-YSANAACGAGGC-3′) and the CaRE1 (5′-CTATTTCGAGGC-3′) CaRF binding sequences (**Figure 5d**), strongly suggesting that the ChIP peak regions contain high affinity binding sites for CaRF.

To characterize CaRF binding to the chCaRE sequences, we first tested the ability of CaRF to bind the specific chCaRE motif (5′-AAAGCGAGGC-3′) found in a CaRF ChIP peak from the promoter of the *Camk2n1* gene (camCaRE, **Figure 5d**). Recombinant CaRF binds the camCaRE as evidenced by retardation of the radiolabeled camCaRE probe by EMSA (**Figure 5b**). This association is fully competed by addition of an excess of unlabeled camCaRE or cCaRE but not by addition of excess unlabeled mutant mCaRE, demonstrating sequence-specific binding of CaRF to the camCaRE probe. Importantly, the association of CaRF with the camCaRE is also strongly competed by a probe representing the chCaRE motif despite the presence of degenerate bases at positions 1,2,4, and 5.

To further understand the relative importance for CaRF binding affinity of specific bases at each position across the chCaRE motif, we changed each base in the camCaRE motif one at a time and tested the ability of the changed motifs to compete CaRF binding to camCaRE by EMSA (**Figure 5c**). Consistent with the evidence that there was little sequence variability in the chCaRE sequence at positions 3, 6, 7, and 8 (**Figure 5a**) switching the base at any of these positions essentially eliminated the ability of the altered oligo to compete the binding of CaRF to the camCaRE (**Figure 5c**). These data reveal that CaRF has a strong requirement for an A at position 3, and GAG at positions 6, 7, and 8 of the chCaRE. Changing the bases at positions 1, 9, or 10 partially impaired the ability of oligos to compete with CaRF **(** **Figure 5c**). These data suggest CaRF has a weak requirement for specific bases at positions 1, 9, and 10, consistent with the fact that the preference for a specific base at these positions in the chCaRE motif was lower than at positions 3, 6, 7, and 8 (**Figure 5a**). Finally, changing the bases of the camCaRE at positions 2, 4, and 5 had a limited effect on the ability of these oligos to compete for the binding of CaRF (**Figure 5c**), indicating that CaRF has little preference for the specific bases at these positions.

By comparing these sequence requirements at each position across the camCaRE to the sequences of the individual chCaRE sites identified in each of the ChIP peaks (**Table S4**), we estimated the likelihood that CaRF would bind by EMSA to each individual chCaRE motif. Strikingly, 45% of the peaks (62/138) contain a chCaRE sequence that we predict would bind with high affinity to CaRF, while an additional 19.5% (27/138) have sequences that we predict would bind with lower affinity. Only 35.5% of the chCaRE sequences (49/138) have sequences that we predict would show no significant affinity for CaRF by EMSA. Whether CaRF may bind to other sequence motifs in these ChIP peaks or whether CaRF may bind these variant chCaRE motifs in collaboration with a binding partner remains unknown. Nonetheless, taken together these data provide strong evidence to suggest that the ChIP peaks represent sites of high affinity CaRF binding to neuronal genomic DNA *in vivo*.

### Functional analysis of CaRF ChIP targets

We next asked whether examination of the set of putative CaRF target genes could suggest potential functions for CaRF in neurons. Statistical analysis of the representation of Gene Ontology (GO) terms for the genes on this list was conducted using GOstat (http://gostat.wehi.edu.au). This analysis revealed two statistically significant overrepresented GO categories (**Table 1**). The first, proline translase activity, contained only two genes from the list of putative CaRF targets and thus was not considered further. By contrast, there are 15 genes from the list of genes with CaRF ChIP peaks that fall into the second category, phosphorous metabolism. 13 of these gene products are kinases or phosphatases that are also included in a closely related GO category, post-translational protein modification. This category approached significance for overrepresentation and contained 4 additional genes from the CaRF ChIP list whose products regulate protein ubiquitination, SUMOylation, and sulfonation. Finally, 6 of the putative CaRF target genes were not part of a defined GO category but caught our attention for their involvement in calcium-dependent signaling events. These genes are interesting in light of our previous observation that the transcriptional activity of CaRF can be modulated in a neuronal and calcium-selective manner [14], because they suggest that CaRF may act not only downstream, but also upstream of calcium signaling in neurons. Taken together these data predict that CaRF may play an important role in regulating neuronal intracellular signaling pathways.

**Table 1:** GOstat analysis of putative CaRF targets.

GO Category	Genes	*p* value
**Proline translase activity**	*Eprs*	0.00956
GO:004827	*Pars2*	
GO:0006433		
**Phosphorous Metabolism**	*Acp1*	0.00956
GO: 0006783	*Atp5d*	
GO:0006796	*Atp6c0e*	
	*Dyrk2*	
	*Epha3*	
	*Epha6*	
	*Ikbke*	
	*Limk2*	
	*Map3k3*	
	*Map4k4*	
	*Mark3*	
	*Prkar1a*	
	*Ptpre*	
	*Ptprs*	
	*Srpk2*	
**Post-translational**	*Chst8*	0.058
**protein modification**	*Fbxl20*	
GO: 0043687	*Park2*	
	*Pias4*	
(also includes 13 genes from the category above)		
**Calcium signaling**	*Cacng2*	N/A
(not a GO category)	*Caly*	
	*Camk2n1*	
	*Camsap1l1*	
	*Camta1*	
	*Syt1*	

**Figure 6:**
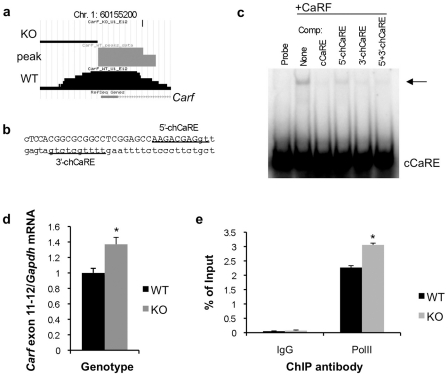
CaRF regulates *Carf* transcription in neurons. a) Primary data from the UCSC genome browser (http://genome.ucsc.edu) showing the CaRF ChIP peak overlapping exon 1 of the *Carf* gene. b) Position of CaRF-binding motifs in the CaRF ChIP peak from the *Carf* gene. Capital letters denote exon 1. The underlined sequences show the two potential CaRF-binding motifs. The more 3′ motif in intron 1 was identified by the PRIORITY motif finder. c) Competition EMSA analysis demonstrates that CaRF can bind both motifs in the *Carf* ChIP peak. Recombinant CaRF was bound to a radiolabeled cCaRE probe in the absence (-) or presence of a 100-fold molar excess of competitor probes. Arrow indicates the CaRF-cCaRE complex. Unlabeled probes used as competitors are listed across the top. d) Expression of *Carf* mRNA in a *Carf* exon 8 KO mouse. Cortical neurons from individual P0 WT or CaRF exon 8 deleted (KO) mice were cultured for 5 days, treated with 1µM TTX overnight, then RNA was harvested for cDNA synthesis and quantitative PCR. *Carf* mRNA was detected with primers against exons 11–12 distal to the deleted region in *Carf*. *Carf* mRNA expression was normalized for expression of *Gapdh* in the same sample to control for sample handling. e) Chromatin immunoprecipitation for RNA polymerase II on the *Carf* promoter. *Carf* promoter DNA co-precipitated with an anti-RNA polymerase II antibody or control IgG was quantitated by Q-PCR, and normalized as a percent of signal in the input DNA. Bars show the mean and error bars show SEM. *p<0.05.

We observed that a CaRF ChIP peak overlaps exon 1 of the *Carf* gene itself (**Figure 6a**), raising the possibility that, like many other eukaryotic transcription factors [30], CaRF might regulate its own expression. In addition to the chCaRE motif identified by the PRIORITY motif finder, we found a second chCaRE-like site within this ChIP peak (**Figure 6b**). Probes containing either of these motifs are capable of competing the binding of recombinant CaRF to the cCaRE in an EMSA, although the PRIORITY-identified site has higher affinity for CaRF (**Figure 6c**). To test whether CaRF might transcriptionally regulate its own expression *in vivo*, we took advantage of the fact that the *Carf* KO mice lack only a single exon of this gene (exon 8)[15]. Although no functional CaRF protein is detected in the KO mice, *Carf* mRNA is still transcribed. Quantitative PCR of *Carf* using primers distal to the exon 8-deleted region reveal that *Carf* mRNA expression is significantly elevated in the absence of CaRF protein (WT = 1±0.059, KO = 1.37±0.089, *n* = 8/genotype, *p* = 0.036; **Figure 6d**). Consistent with the possibility that this enhanced *Carf* RNA expression is due to increased transcription of the *Carf* gene, as opposed to a change in RNA stability, we also find an increase in RNA polymerase II association with the *Carf* promoter in *Carf* exon 8 KO mice (WT = 2.26±0.06, KO = 3.05±0.06, *n* = 2/genotype, *p* = 0.0125, **Figure 6e**).

**Figure 7:**
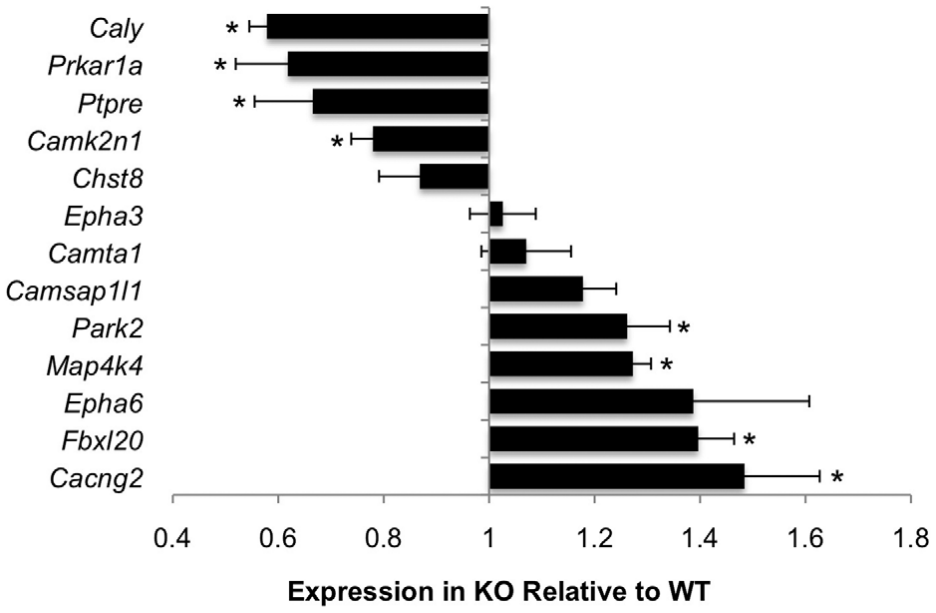
Altered expression of a subset of the putative CaRF target genes in *Carf* knockout mice. RNA from P0 cortex of *Carf* WT or KO was processed for quantitative PCR using primers against a subset of the putative CaRF target genes. In each case, mRNA expression was normalized for expression of *Gapdh* in the same sample to control for sample handling. Data are displayed as expression in KO brains relative to expression in WT brains. A value of 1 indicates no difference in expression, whereas values less than 1 indicate reduced expression in KO compared with WT, and values greater than 1 indicate increased expression in KO compared with WT. *n* = 4–6 for each genotype. Bars show the mean and error bars represent S.E.M. *p<0.05 for KO compared with WT.

To determine whether other genes that contain CaRF ChIP peaks also show altered expression in the absence of functional CaRF protein, we compared the expression of 13 of the genes from **Table 1** between the brains of newborn *Carf* WT and KO mice (**Figure 7**). Expression of 8 of these gene products was significantly different between WT and KO brains, with 4 genes showing significantly higher expression in WT compared with KO, while 4 showed significantly lower expression in WT compared with KO. These data demonstrate that CaRF is required for proper expression of a least a subset of the gene products that neighbor the CaRF ChIP peaks.

## Discussion

CaRF was first cloned in a yeast one-hybrid screen based on its ability to bind to the CaRE1 calcium-response element of the *Bdnf* gene [14]. Further characterization verified that CaRF is a nuclear protein and that overexpression of CaRF drives CaRE1-dependent transcription, however mapping of the domain of CaRF required for binding to CaRE1 revealed that this region shares no sequence homology with any known family of DNA binding domain proteins. This was intriguing because most transcription factors are part of large families defined by the homology of their DNA binding domains, whereas our data suggested that CaRF is unique.

To gain more insight into the importance of the CaRF DNA binding domain, we first asked whether this region has been conserved over evolutionary time. We identified conserved CaRF orthologs from 35 vertebrates, a deuterostome, and the cnidarian *Nematostella vectensis*. We did not find any evidence for CaRF orthologs in plant genomes, suggesting that CaRF co-evolved with the development of animal-specific functions, which include processes such as cell-cell adhesion, cell signaling, and synaptic transmission. Nor, intriguingly, were any orthologs identifiable in the completed genomes of *C. elegans* or *D. melanogaster* despite the fact that these organisms evolved after *N. vectensis*. The reasons for this are unclear, although it has been observed that the genome of *N. vectensis* has a gene repertoire, intron-exon structure and gene linkage map that is more similar to that of vertebrates than to flies or nematodes [29]. Importantly we find only one gene with any similarity to CaRF in each species, confirming that CaRF is unlikely to be part of a larger transcription factor family. By aligning these sequences, we show that the DNA binding domain of CaRF has been highly conserved across hundreds of millions of years of evolution, strongly suggesting that CaRF has a conserved function as a DNA binding protein. Given our evidence that CaRF binds directly to DNA, and the fact that DNA binding domains are otherwise some of the best understood protein structures, it will of great interest in the future to determine the structure of the CaRF DNA binding sequence to understand this novel domain.

We took two complementary approaches to identifying CaRF binding sites. First we conducted a PCR-assisted binding site selection screen *in vitro* which identified a 12bp high-affinity CaRF-binding motif (cCaRE). In an independent set of experiments, we identified a highly similar motif (the 10bp chCaRE) when we aligned the fragments of neuronal genomic DNA that specifically coimmunoprecipitated with CaRF by ChIP. The chCaRE motif was found in 78% of the 176 CaRF ChIP peaks, and by testing the requirement for each degenerate base across the chCaRE motif we provide evidence that CaRF is likely to show affinity for at least 64.5% of the variant chCaRE motifs *in vitro*. These data raise the question of whether CaRF also directly associates with the other 35.5% of the ChIP peaks that contain base changes in key positions within the chCaRE motif, as well as those that lack a sequence with any similarity to the cCaRE.

Transcription factors vary in their requirement for a consensus motif to guide their selection of genomic binding sites [5]. For example, consensus binding motifs are highly enriched in genomic binding sites for the transcriptional repressor REST [7], whereas other factors, including the neuronal activity-regulated transcription factor CREB, are frequently found bound to regions that lack a consensus binding sequence [8]. In the case of CaRF, one possibility is that in addition to cCaRE-like sequences, CaRF may directly bind additional distinct sequence motifs that were not identified in our binding site selection screen. A recent comprehensive analysis of DNA binding preferences conducted for a large group of transcription factors revealed that approximately half of these factors recognized multiple distinct sequence motifs, indicating that DNA binding preferences of transcription factors may be more flexible than previously realized [31]. It is also possible that the association of CaRF with peaks that lack consensus binding sites is indirect. Protein-protein interactions can recruit transcription factors to regions that lack consensus binding motifs either through an association that depends solely on the binding of the interacting factor to DNA, or by cooperative binding between the transcription factor of interest and the interacting factor in a manner that changes the affinity of the transcription factor for non-consensus DNA motifs [5]. It would be particularly interesting if CaRF were recruited to consensus and non-consensus binding sites through distinct mechanisms, as this would imply a potential means to differentially regulate the expression of subsets of CaRF target genes.

GOstat analysis of the genes that neighbor the CaRF ChIP peaks revealed overrepresentation of gene products involved in signaling pathways, suggesting the possibility that CaRF-dependent transcription may modulate intracellular states in neurons. Interestingly, the known functions of these gene products suggest possible mechanisms through which CaRF could contribute to experience-dependent synaptic development and plasticity. For example the Eph family receptor tyrosine kinases *Epha3* and *Epha6* are regionally expressed in the developing brain, where they play important roles in neural patterning [32]. Within the visual system, EphA signaling has been shown to cooperate with neural activity in the refinement of the synaptic connections that are required for the establishment of topographic maps in both the thalamus and the visual cortex during development [33], [34]. In addition, among the putative CaRF target genes that are related to calcium signaling, the proteins encoded by *Caly* (Calcyon) and *Cacng2* (Stargazin) contribute to the regulation of AMPA-type glutamate receptor trafficking to synapses [35], [36] suggesting a possible mechanism by which CaRF-dependent transcription could modulate the strength of excitatory synapses. These data are particularly exciting in light of our findings that adult *Carf* KO mice show abnormalities in several tests of learning and memory [15]. Specifically these mice display impaired extinction of context-dependent fear conditioning as well as reduced remote memory retention in a novel object recognition task. Performance in both of these memory tests is thought to depend on experience-dependent changes in cortical synaptic function [37], [38]. Whether activity-dependent plasticity of glutamatergic synapses is altered in the *Carf* knockout mice, and whether any the gene products identified in this study contribute to the memory phenotypes in these mice, will be interesting questions to explore.

Despite the evidence for the enrichment of functional pathways among the putative CaRF targets, it remains possible that our list of genomic CaRF binding sites is incomplete. To ensure the specificity of ChIP using the anti-CaRF antibody, we sequenced ChIP libraries made from both *Carf* WT and KO neurons then developed a statistical method to identify only those genomic regions significantly enriched in the pulldown from the WT cells. This method of analysis yielded a relatively small number of peaks compared with other genome-scale ChIP studies [7], [39], [40], suggesting that by setting our parameters to define a stringent threshold for peak detection we may have increased the likelihood of false negatives. For example, we did not find a CaRF ChIP peak in promoter IV of the *Bdnf* gene, which could indicate either that CaRF was not bound to *Bdnf* CaRE1 under the conditions used in this study or that this interaction was undetectable for technical reasons. In addition, despite the evidence that the transcriptional activity of CaRF can be acutely enhanced by the activation of L-type voltage gated calcium channels in neurons [14], we did not find extensive overlap between the list of putative CaRF target genes (**Table S4**) and several large published sets of neuronal activity-dependent genes [19], [41], [42].

Nonetheless, we have demonstrated that at least a subset of the putative CaRF target genes show altered expression in the brains of *Carf* KO mice, demonstrating that CaRF expression is important for their proper regulation. It is interesting that whereas in heterologous overexpression assays CaRF acts as a CaRE1-dependent transcriptional activator of a *Bdnf* promoter IV-luciferase reporter [14], the data presented in **Figure 6d** and **Figure 7** demonstrate both up- and down-regulation of genes with CaRF ChIP peaks in the *Carf* KO mice. Although loss of CaRF may indirectly elevate gene expression, because the genes we have assayed here directly neighbor sites of CaRF binding in vivo, these data raise the possibility that CaRF acts as a direct repressor of a subset of its target genes. The functions of many transcriptional regulators are highly context dependent, and can be influenced by cell type [43], post-translational modifications [44], [45], protein-protein interactions [46], as well as the local chromatin environment [47]. The molecular mechanisms by which CaRF regulates transcription are not fully understood, however our previous overexpression experiments indicated that the C-terminal region of CaRF was required for its ability to activate the *Bdnf* promoter IV reporter plasmid [14]. In this context it is interesting that in addition to producing full-length *Carf* transcripts, we have found that the *Carf* locus also encodes a C-terminally truncated *Carf* variant through the use of an alternative polyadenylation site in the intron just following the exons that encode the DNA binding domain [15]. This variant is expressed in brain and is predicted to bind DNA but not to activate transcription. Thus the short variant of CaRF could potentially act as an endogenous dominant negative of CaRF-dependent transcription. Our antibody was raised against the complete CaRF protein and will immunoprecipitate both forms of CaRF, and importantly both the short and the long variants of CaRF are absent in our KO mice [15]. However whether different CaRF variants are recruited differentially to different regions of the genome, and whether either form of CaRF can actively repress transcription remains to be tested. In conclusion, by continuing to combine the analysis of gene expression in our *Carf* KO mouse with the identification of genomic binding sites of CaRF presented here we expect these data will provide a crucial starting point for a detailed study of the molecular mechanisms that regulate CaRF-dependent transcription in neurons.

## Supporting Information

Table S1 Oligonucleotides used in this study.
(0.07 MB DOC)

Table S2 CaRF orthologs. CaRF orthologs were identified by BLAST search for translated nucleotide sequences with high similarity to Mus musculus CaRF. Orthologs from 37 species were identified, and accession numbers are listed below. ENS sequences are from Ensembl (http://www.ensembl.org) and XM, XR, and NR sequences are from NCBI (http://www.ncbi.nlm.nih.gov/entrez).
(0.04 MB DOC)

Table S3 Identification of a high affinity CaRF binding motif by PCR-assisted site selection. Oligos selected after four rounds of coimmunoprecipitation with CaRF were cloned in the vector pBluescript and sequenced. 62 sequences were aligned and the best fitted 16bp motif is shown for each clone. Capital letters indicate bases within the random 16mer sequence and lower case letters indicate the flanking sequences.
(0.04 MB DOC)

Table S4 Genomic locations of CaRF ChIP peaks and identification of a conserved in vivo CaRF binding motif. Peak files derived from the CaRF ChIP were loaded into the UCSC browser to analyze genomic location. Location denotes the chromosomal position of each peak. The peak start and end numbers are in reference to build 37 of the Mus musculus genome (mm9, July 2007; http://genome.ucsc.edu). The nearest annotated gene within 10kB of the ChIP peak is listed under Gene Symbol. The Motif column shows the sequence of the common 10bp motif found by PRIORITY analysis of the ChIP peak sequences. The sequences not included in the analysis are marked “too short”, and sequences analyzed that did not contain a conserved motif are marked “none.” Finally based on in vitro analysis of CaRF's tolerance for base pair changes across the motif as shown in Figure 5c, we predicted the affinity of CaRF for each of the motif sequences by EMSA. Motifs with base pair changes in positions 3, 6, 7, or 8 are denoted as “Low” affinity, motifs with base pair changes in positions 1, 9, or 10 are denoted “Medium” affinity, and the remaining motifs are “High” affinity. The 60 peaks highlighted in gray are within 1kB of a transcriptional start site and were used for the positional analysis of CaRF binding sites in Figure 4.
(0.27 MB DOC)
