# Supplementary material for: Genome-Wide Identification of Calcium-Response Factor (CaRF) Binding Sites Predicts a Role in Regulation of Neuronal Signaling Pathways
Source: PLoS One. 2010 May 27;5(5):e10870. doi: 10.1371/journal.pone.0010870 (PMC2877716; doi:10.1371/journal.pone.0010870)
Supplement: Table S1 — Oligonucleotides used in this study. (0.07 MB DOC) [file pone.0010870.s001.doc]

| **EMSA Oligonucleotide Binding Probes (and Reverse Complement)** |
| --- |
| CaRE1: 5’-GAG TGT CTA TTT CGA GGC AGA GGA GG-3’ |
| cCaRE: 5’-GAG TGT YCA RAA CGA GGC AGA GGA GG-3’ |
| mCaRE1: 5’-GAG TGT CTT CCG CCA GGC AGA GGA GG-3’ |
| camCaRE: 5’-TCA AAG CGA GGC AGA-3’ |
| chCaRE: 5’-TCR RAR YGA GGC AGA-3’ |
| CaRF1: 5’-GCC TCG GAG CCA AGA CGA GGT TGA GTA-3’ |
| CaRF2: 5’-TGA GTA GTC TCG TTT TGA ATT TTC TCC-3’ |
| CaRF1+2: 5’-GCC TCG GAG CCA AGA CGA GGT TGA GTA GTC TCG TTT TGA ATT TTC TCC-3’ |
| **Selection Screen Oligos** |
| 5’-CAGGTCAGTTCAGCGGATCCTGTCGNNNNNNNNNNNNNNNNGAGGCGAATTC  AGTGCAACTGCAGC-3’ |
| PCR F: 5’-GCTGCAGTTGCACTGAATTCGCCTC-3’ |
| PCR R: 5’-CAGGTCAGTTCAGCGGATCCTGTCG-3’ |
| **Q-PCR Primers for RNA expression** |
| Mouse/Rat *Gapdh* F: 5’- CATGGCCTTCCGTGTTCCT-3’ |
| Mouse/Rat *Gapdh* R: 5’- TGATGTCATCATACTTGGCAGGTT-3’ |
| Mouse *Carf* exon 11F: 5’-CCGCAAGTAGCGCATAAGAT-3’ |
| Mouse *Carf* exon 12R: 5’-TCCAGTCCTCAAGGATTTCTG-3’ |
| Mouse *Cacng2* F: 5’-GAGGGCCTCGAGTATCTTCC-3’ |
| Mouse *Cacng2* R: 5’-TAGAAGGACCAGCCGTAGGA-3’ |
| Mouse *Caly* F: 5’-GACCAGCCAAAGAAGTTTGC-3’ |
| Mouse *Caly* R: 5’-CTTGTGCCGAAGTAGGAAGC-3’ |
| Mouse *Camk2n1* F: 5’-TGCAGGACACCAACAACTTC-3’ |
| Mouse *Camk2n1* R: 5’-AGCCCGCCACTCTTCTTATT-3’ |
| Mouse *Camsap1l1* F: 5’-AAGGCCCTCCAGTCTCTAGC-3’ |
| Mouse *Camsap1l1* R: 5’-AAGTTGTGGATGCGTTTTCC-3’ |
| Mouse *Camta1* F: 5’-CCATGAGCCCTCCAGAAATA-3’ |
| Mouse *Camta1* R: 5’-GACCTAGGGCTCTGCTTCCT-3’ |
| Mouse *Chst8* F: 5’-ACGTGCCTTTTACACCCAAG-3’ |
| Mouse *Chst8* R: 5’-GTGTGCCCTTTTCTGTGGAT-3’ |
| Mouse *Epha3* F: 5’-GAGACAGTATGCCGCAGTCA-3’ |
| Mouse *Epha3* R: 5’-GCCTCTTGCTCTCAAAATGG-3’ |
| Mouse *Epha6* F: 5’-CTATTCCTCCACGAGGTCCA-3’ |
| Mouse *Epha6* F: 5’-CTGCAGTGGCTATGACCAGA-3’ |
| Mouse *Fbxl20* F: 5’-CCAGCTGGAGGTGATAGAGC-3’ |
| Mouse *Fbxl20* R: 5’-GAGTGACAGGGGCGAAGTAG-3’ |
| Mouse *Map4k4* F: 5’-TGGTGTGCTACGAAGACGAG-3’ |
| Mouse *Map4k4* R: 5’-GTCTCTGAGCCCTTTTGTGC-3’ |
| Mouse *Park2* F: 5’-TGGAAAGCTCCGAGTTCAGT-3’ |
| Mouse *Park2* R: 5’-CCTTGTCTGAGGTTGGGTGT-3’ |
| Mouse *Prkar1a* F: 5’-TGGAGAGCTGGCTTTGATTT-3’ |
| Mouse *Prkar1a* R: 5’-TGCATCGGCTACTGTGAGAC-3’ |
| Mouse *Ptpre* F: 5’-CTGGCTCACACAGTTGAGGA-3’ |
| Mouse *Ptpre* R: 5’-GGGCTGTTTGAAGGTAACCA-3’ |
| **Q-PCR Primers for ChIP on the *Carf* Gene** |
| F: 5’-ACG AGT CGG AAG GGA ACT G-3’ |
| R: 5’-CCG TAA GCA GAA GGG AGA AA-3’ |
