# Supplementary material for: Genome-Wide Identification of Calcium-Response Factor (CaRF) Binding Sites Predicts a Role in Regulation of Neuronal Signaling Pathways
Source: PLoS One. 2010 May 27;5(5):e10870. doi: 10.1371/journal.pone.0010870 (PMC2877716; doi:10.1371/journal.pone.0010870)
Supplement: Table S2 — CaRF orthologs. CaRF orthologs were identified by BLAST search for translated nucleotide sequences with high similarity to Mus musculus CaRF. Orthologs from 37 species were identified, and accession numbers are listed below. ENS sequences are from Ensembl (http://www.ensembl.org) and XM, XR, and NR sequences are from NCBI (http://www.ncbi.nlm.nih.gov/entrez). (0.04 MB DOC) [file pone.0010870.s002.doc]

| **Genus and species** | **Accession #** |
| --- | --- |
| *Bos taurus* | NM_001032294.1 |
| *Brachiostoma floridae* | XM_002220714.1 |
| *Canis familiarus* | XM_536037.2 |
| *Cavia porcellus* | ENSCPOG00000008827 |
| *Choloepus hoffmanni* | ENSCHOG00000005826 |
| *Danio rerio* | XM_678321.3 |
| *Dasypus novemcinctus* | ENSDNOG00000025534 |
| *Dipodomys ordii* | ENSDORG00000006614 |
| *Echinops telfairi* | ENSETEG00000018695 |
| *Equus caballus* | XM_001497530.2 |
| *Erinaceus europaeus* | ENSEEUG00000002496 |
| *Gallus gallus* | XM_421946.2 |
| *Gorilla gorilla* | ENSGGOG00000005406 |
| *Homo sapiens* | NM_001104586.1 |
| *Loxodonta africana* | ENSLAFG00000017289 |
| *Macaca mulatta* | XR_012691.1 |
| *Microcebus murinus* | ENSMICG00000011943 |
| *Monodelphis domestica* | XM_001371558.1 |
| *Mus musculus* | NM_139150.4 |
| *Myotis lucifugus* | ENSMLUG00000007015 |
| *Nematostella vectensis* | XM_001634671.1 |
| *Ochotona princeps* | ENSOPRG00000008406 |
| *Ornithorhynchus anatinus* | ENSOANG00000011095 |
| *Oryctolagus cuniculus* | ENSOCUG00000005335 |
| *Pan troglodytes* | NM_001106915.1 |
| *Pongo pygmaeus* | ENSPPYG00000013090 |
| *Procavia capensis* | ENSPCAG00000008043 |
| *Pteropus vampyrus* | ENSPVAG00000005257 |
| *Rattus norvegicus* | NM_001106915.1 |
| *Sorex araneus* | ENSSARG00000012208 |
| *Spermophilus tridecemlineatus* | ENSSTOG00000001585 |
| *Sus scrofa* | XM_001924977.1 |
| *Taeniopygia guttata* | XM_002187657.1 |
| *Tarsius syrichta* | ENSTSYG00000004672 |
| *Tupaia belangeri* | ENSTBEG00000004957 |
| *Tursiops truncatus* | ENSTTRG00000006016 |
| *Vicugna pacos* | ENSVPAG00000009501 |
