# Supplementary material for: Genome-Wide Identification of Calcium-Response Factor (CaRF) Binding Sites Predicts a Role in Regulation of Neuronal Signaling Pathways
Source: PLoS One. 2010 May 27;5(5):e10870. doi: 10.1371/journal.pone.0010870 (PMC2877716; doi:10.1371/journal.pone.0010870)
Supplement: Table S3 — Identification of a high affinity CaRF binding motif by PCR-assisted site selection. Oligos selected after four rounds of coimmunoprecipitation with CaRF were cloned in the vector pBluescript and sequenced. 62 sequences were aligned and the best fitted 16bp motif is shown for each clone. Capital letters indicate bases within the random 16mer sequence and lower case letters indicate the flanking sequences. (0.04 MB DOC) [file pone.0010870.s003.doc]

| GGATCCATATCgaggc | TGTGCGAAACCgaggc |
| --- | --- |
| TTTACGAAAGCgaggc | GTAACCAGGACgaggc |
| GCTCTGAAAACgaggc | AAAATCATAACgaggc |
| GGTTCCATAACgaggc | ACCTTCAGAACgaggc |
| TGGGCGGAAACgaggc | GTTCCCACATCgaggc |
| GAATCCACATCgaggc | GGAAAAACTCGgaggc |
| TACGCCAGAACgaggc | TGGTCGAGATCgaggc |
| CTGATCAAAGCgaggc | tgtcgCAAAACGAGGT |
| GTCATCAGAACgaggc | TGGTCCAAAACgaggc |
| TGCCTCAGAACgaggc | CGACCCAGAACgaggc |
| tgtcgGAAAGCGAGGC | ATTACCAGAACgaggc |
| TTAACCAGAACgaggc | AGCCCCAAAACgaggc |
| tgtcgCATAACGAGGC | TCAGTCATAACgaggc |
| ctgtcgAAAGCGAGGC | CGTATCAGAACgaggc |
| GGGCTCAAAACgaggc | TTCGCGAGAACgaggc |
| GATCTGAAACCgaggc | CGGATGAAAACgaggc |
| CGTGCCAGATCgaggc | CGCTCCAGAACgaggc |
| CAGCCGAAACCGAGGC | CGTGTCAAAACgaggc |
| ctgtcgGCTGTGAGGC | tcgATCATACCGAGGC |
| AACTCGAGAACgaggc | GCTGCGAGAACgaggc |
| CGCTCCACATCgaggc | ATGGCGAAAACgaggc |
| AATTTCAGAGCgaggc | GGGGTCAGAACgaggc |
| GGGGTGGTAACgaggc | GACTTCAGAACgaggc |
| CAAGTGAAAACgaggc | GGTCCCAAAACgaggc |
| GACCCAAACCAgaggc | ACTCTCAGATCgaggc |
| AGAGCGAGAACGAGGC | AACTTCATAACgaggc |
| CGATTCAGAACgaggc | CGGCCCAGAACgaggc |
| TGTATGAAAACgaggc | gGAGTGGAAACGAGGC |
| AGCGCGAAAACgaggc | TTCGCGAAAGCgaggc |
| CTATTCAAAACgaggc | TTACCAAAAACgaggc |
| TCGGCCACAACgaggc | AATACCAAAGCgaggc |
