# Supplementary material for: Genome-Wide Identification of Calcium-Response Factor (CaRF) Binding Sites Predicts a Role in Regulation of Neuronal Signaling Pathways
Source: PLoS One. 2010 May 27;5(5):e10870. doi: 10.1371/journal.pone.0010870 (PMC2877716; doi:10.1371/journal.pone.0010870)
Supplement: Table S4 — Genomic locations of CaRF ChIP peaks and identification of a conserved in vivo CaRF binding motif. Peak files derived from the CaRF ChIP were loaded into the UCSC browser to analyze genomic location. Location denotes the chromosomal position of each peak. The peak start and end numbers are in reference to build 37 of the Mus musculus genome (mm9, July 2007; http://genome.ucsc.edu). The nearest annotated gene within 10kB of the ChIP peak is listed under Gene Symbol. The Motif column shows the sequence of the common 10bp motif found by PRIORITY analysis of the ChIP peak sequences. The sequences not included in the analysis are marked “too short”, and sequences analyzed that did not contain a conserved motif are marked “none.” Finally based on in vitro analysis of CaRF's tolerance for base pair changes across the motif as shown in Figure 5c, we predicted the affinity of CaRF for each of the motif sequences by EMSA. Motifs with base pair changes in positions 3, 6, 7, or 8 are denoted as “Low” affinity, motifs with base pair changes in positions 1, 9, or 10 are denoted “Medium” affinity, and the remaining motifs are “High” affinity. The 60 peaks highlighted in gray are within 1kB of a transcriptional start site and were used for the positional analysis of CaRF binding sites in Figure 4. (0.27 MB DOC) [file pone.0010870.s004.doc]

| **Location** | **Peak Start** | **Peak End** | **Gene Symbol** | **Motif** | **Predicted Affinity** |
| --- | --- | --- | --- | --- | --- |
| 1qA2 | 10028060 | 10028250 | *Cops5* | GAAACGAGAA | Medium |
| 1qA5 | 24622670 | 24622690 | *Atpase6 (pred.)* | Too Short | n/a |
| 1qB | 39999940 | 40000000 | *Map4k4* | AGAATGAGGC | High |
| 1qC1.3 | 58449175 | 58449200 | *Bzw1* | AGAGAGAGAG | Medium |
| 1qC2 | 60155120 | 60155210 | *Carf* | AAAACGAGAC | Medium |
| 1qC5 | 84836140 | 84836270 | *Fbxo36* | GAACTGAGAG | Medium |
| 1qE1.2 | 104249870 | 104249940 | *None* | GGAGAGCCAT | Low |
| 1qE4 | 133157070 | 133157180 | *Ikbke* | AGATCGAGGC | High |
| 1qE4 | 135027770 | 135027800 | *Ppp1r15b* | AGGGTGAGGC | Low |
| 1qE4 | 138242770 | 138242880 | *Camsap1l1* | GGATAGGGGC | Low |
| 1qH4 | 180248800 | 180248910 | *2310005N03RIK* | GGAACGAGGC | High |
| 1qH5 | 187186710 | 187186940 | *Eprs* | GGAGTGGGGC | Low |
| 4qA1 | 6723210 | 6723250 | *Tox* | AAACTAAGAC | Low |
| 4qA1 | 11413080 | 11413090 | *1110037F02RIK* | Too Short | n/a |
| 4qC1 | 64259600 | 64259720 | None | GAGCCTATTT | Low |
| 4qC7 | 106323510 | 106323600 | *Pars2* | AGAGCGAGGC | High |
| 4qD2.2 | 123876130 | 123876260 | None | AGAACGAGGC | High |
| 4qD3 | 136158690 | 136158810 | *Aof2* | GAAGTCAGGC | Low |
| 4qD3 | 137982920 | 137983040 | *Mul1* | AAATCGAGGC | High |
| 4qD3 | 138010240 | 138010330 | *Camk2n1* | AAAGCGAGGC | High |
| 4qD3 | 140616600 | 140616720 | *Crocc* | GGACCGAGGC | High |
| 4qE2 | 150951050 | 150951120 | *Camta1* | CACTTCCACC | Low |
| 4qE2 | 152732550 | 152732560 | *Ajap1* | Too Short | n/a |
| 4qE2 | 154469460 | 154469640 | *Morn1* | AAAGTGAGGC | High |
| 5qA3 | 23122281 | 23122310 | *Srpk2* | GGGATTAGGG | Low |
| 5qA3 | 24191211 | 24191290 | *Nub1* | AGACCGAGTT | Medium |
| 5qC3.2 | 72950981 | 72950990 | *Nfxl1* | Too Short | n/a |
| 5qE2 | 93270571 | 93270640 | *Shroom3* | GAAGGAAGAC | Low |
| 5qE5 | 107231601 | 107231660 | None | AGGATCAGGT | Low |
| 5qF | 116182651 | 116182760 | *Ccdc64* | AAAACGACCG | Low |
| 5qF | 116182900 | 116182960 | *Ccdc64* | TGAGTCAGGG | Low |
| 5qG2 | 143619521 | 143619620 | *Fbxl18* | AGATAGAGGC | High |
| 7qA1 | 3596700 | 3596810 | *Cnot3* | AGAATGAGGC | High |
| 7qA3 | 25794250 | 25794320 | *Atp1a3* | AGAACGAGGC | High |
| 7qA3 | 25861961 | 25862070 | *Zfp574* | AGAGGAAGAA | Low |
| 7qA3 | 25862180 | 25862370 | *Zfp574* | AGAGCGAGGC | High |
| 7qB1 | 35457131 | 35457180 | *Chst8* | AAACAGAGGC | High |
| 7qB2 | 38635131 | 38635180 | None | AAAATGAGGC | High |
| 7qB4 | 51592261 | 51592320 | None | GCACACAGAC | Low |
| 7qD3 | 91300841 | 91300860 | *Fam108c* | Too Short | n/a |
| 7qD3 | 91743061 | 91743170 | *Fah* | ATACTGAGTC | Medium |
| 7qE1 | 103945161 | 103945170 | *Odz4* | Too Short | n/a |
| 7qF1 | 120909801 | 120909830 | *Spon1* | None | n/a |
| 7qF3 | 142757831 | 142757940 | *Ptpre* | TGAACGACAC | Low |
| 7qF4 | 147268381 | 147268570 | *Caly* | AGAGCGAGGC | High |
| 7qF5 | 148246481 | 148246580 | *B4galnt4* | AGAGGAAGGC | Low |
| 8qA1.1 | 4166021 | 4166120 | *Evi5l* | GGAATGAGGT | Medium |
| 8qA1.1 | 6096360 | 6096480 | None | AAAGAGAGGC | High |
| 8qA1.1 | 11114711 | 11114760 | None | AAAGTAAGAT | Low |
| 8qA2 | 28109771 | 28109780 | None | Too Short | n/a |
| 8qA2 | 28622431 | 28622520 | None | AGACAGGGAC | Low |
| 8qA4 | 41966351 | 41966360 | *Slc7a2* | Too Short | n/a |
| 8qD1 | 97856191 | 97856260 | *Aa960436* | TGGTCGAGGC | Low |
| 8qD3 | 106585971 | 106586080 | *Cdh5* | AGAGTGAGGC | High |
| 8qD3 | 109526521 | 109526630 | *Sntb2* | GAAATGAGGC | High |
| 8qD3 | 112302441 | 112302500 | *Ap1g1* | TAGCTCAGAC | Low |
| 8qE1 | 116600701 | 116600840 | *Nudt7* | AAAAAGAGGC | High |
| 8qE2 | 129061021 | 129061270 | None | AGAACGAGGC | High |
| 9qA1 | 3019011 | 3019090 | None | TGAAAAAGGT | Low |
| 9qA1 | 3022240 | 3022300 | None | ATCGCGAGGA | Low |
| 9qA1 | 3026530 | 3026600 | None | AAAATGAGAA | Medium |
| 9qA1 | 3032690 | 3032800 | None | AAAATGAGAA | Medium |
| 9qA1 | 3038040 | 3038150 | None | GGAATAAGGC | Low |
| 9qA1 | 13422751 | 13422760 | *Maml2* | Too Short | n/a |
| 9qA1 | 13631411 | 13631560 | *Cep57* | AGACCGAGGC | High |
| 9qA5.1 | 42338221 | 42338330 | *Grik4* | AGAGCGAGGC | High |
| 9qA5.3 | 51573031 | 51573050 | *Arhgap20* | Too Short | n/a |
| 9qB | 62284481 | 62284510 | *Coro2b* | None | n/a |
| 9qC | 66891781 | 66891880 | *Tpm1* | AGAGCGAGGC | High |
| 9qD | 74823861 | 74823900 | *BCO31353* | GACGCGGGGC | Low |
| 9qE3.1 | 88417601 | 88417900 | *4930422I07RIK* | AGATCGAGGC | High |
| 9qF2 | 108686051 | 108686160 | *Ip6k2* | GGAGCGAGGT | Medium |
| 10qA4 | 30320501 | 30320620 | *Trmt11* | GGGATGAGGC | Low |
| 10qC1 | 79604061 | 79604170 | *Atp5d* | AGAGCGAGGC | High |
| 10qC1 | 80630931 | 80631261 | *Pias4* | AAAACGAGGC | High |
| 10qC1 | 80959131 | 80959320 | *Ncln* | AAAACGAGGC | High |
| 10qC3 | 95653350 | 95653440 | None | AGAGAAAGGA | Low |
| 10qC3 | 95952331 | 95952380 | None | AGAGTGAGGT | Medium |
| 10qD1 | 107989731 | 107989770 | *Syt1* | AAAACGAGGC | High |
| 10qD2 | 118393611 | 118393710 | *Dyrk2* | AGGCAGTGCT | Low |
| 11qA1 | 3054271 | 3054330 | *Sfi1* | TTAATGAGTC | Medium |
| 11qA1 | 3073790 | 3073800 | *Sfi1* | Too short | n/a |
| 11qA1 | 3083460 | 3083510 | *Sfi1* | ATATGGAGGC | High |
| 11qA1 | 3099471 | 3099500 | *Eif4enif1* | TGAGACAGGG | Low |
| 11qA1 | 3230811 | 3230880 | *Pik3ip1* | TAACAGGGGC | Low |
| 11qA1 | 3271421 | 3271630 | *Limk2* | GGAGCGAGGC | High |
| 11qA4 | 31900531 | 31900540 | *Nsg2* | Too Short | n/a |
| 11qA4 | 33063281 | 33063460 | *Npm1* | AGAGCGAGGC | High |
| 11qA5 | 42815311 | 42815450 | None | AGAATGAGGC | High |
| 11qB1.3 | 59661501 | 59661520 | *Nt5m* | Too Short | n/a |
| 11qD | 96777711 | 96777920 | *Cdk5rap3* | GAACTGAGGC | High |
| 11qD | 98010901 | 98011000 | *Fbxl20* | GGAGCGGCGT | Low |
| 11qD | 100481091 | 100481110 | *Dnajc7* | Too Short | n/a |
| 11qE1 | 105945921 | 105945950 | *Map3k3* | None | n/a |
| 11qE1 | 109509981 | 109510090 | *Prkar1a* | AGAACGAGGC | High |
| 11qE2 | 116135651 | 116135670 | *Srp68* | Too Short | n/a |
| 11qE2 | 117277011 | 117277040 | None | None | n/a |
| 11qE2 | 118207221 | 118207230 | *Timp2* | Too Short | n/a |
| 12qA1.1 | 8554260 | 8554400 | *Slc7a15* | GAACCGAGGC | High |
| 12qA1.1 | 13991080 | 13991200 | None | AAAGTGAGGC | High |
| 12qA1.2 | 21292120 | 21292300 | *Itgb1bp1* | AGAACGAGGT | Medium |
| 12qA1.3 | 21423470 | 21423500 | *Ywhaq* | Too Short | n/a |
| 12qA2 | 31575060 | 31575170 | *Acp1* | AGAGTGAGGC | High |
| 12qB1 | 40685061 | 40685160 | None | AGAACGAGGC | High |
| 12qC1 | 57642261 | 57642290 | *Nkx2-1* | None | n/a |
| 12qC2 | 68160451 | 68160470 | *Mdga2* | Too Short | n/a |
| 12qD1 | 82379321 | 82379330 | *Slc8a3* | Too Short | n/a |
| 12qF1 | 108167591 | 108167731 | None | AACTCGAGGA | Low |
| 12qF1 | 112797951 | 112798010 | *Mark3* | AGACCGAGGC | High |
| 13qA3.3 | 34138361 | 34138510 | *Bphl* | AGAGTGAGGC | High |
| 13qA4 | 43662961 | 43663020 | *Ccdc90a* | AAAGCGAGGC | High |
| 13qB1 | 57361491 | 57361510 | *Spock1* | Too Short | n/a |
| 13qC3 | 81850001 | 81850110 | *Polr3g* | CCAGAGTGAG | Low |
| 14qA1 | 11074391 | 11074500 | *Fhit* | AAAATGAGGC | High |
| 14qA3 | 21208101 | 21208290 | *Dnajc9* | AGAGCGAGGC | High |
| 14qA3 | 26046141 | 26046270 | None | AGAGCGAGTC | High |
| 14qB | 34734051 | 34734180 | *Gdf10* | GAAATGAGGC | High |
| 14qC1 | 47713241 | 47713310 | *Samd4* | AAACACAGGC | Low |
| 14qC2 | 52930711 | 52930760 | *Sall2* | GTAAAAATAC | Low |
| 14qC3 | 56262391 | 56262400 | *Tm9sf1* | Too Short | n/a |
| 14qE2.2 | 99445491 | 99445530 | *2410129H14Rik* | AGAGCGAGGA | Medium |
| 15qB1 | 25221201 | 25221280 | None | TGAGAGGGGC | Low |
| 15qB1 | 25914121 | 25914190 | *Zfp622* | TAAGAGAGGG | Medium |
| 15qB3.2 | 44259341 | 44259480 | *Nudcd1* | GAACTGAGGC | High |
| 15qD2 | 66508301 | 66508400 | *Tg* | AGATTGAGGC | High |
| 15qD3 | 74917221 | 74917290 | None | AGATGGAGGT | Medium |
| 15qD3 | 75396561 | 75396610 | *Ly6h* | AGACCGAGGC | High |
| 15qD3 | 75900211 | 75900330 | *Scrib* | AGAGCGAGGC | High |
| 15qE1 | 77950681 | 77950760 | *Cacng2* | AGAAGGAGGC | High |
| 15qE1 | 78893321 | 78893420 | *Ankrd54* | AGAGTGCGGG | Low |
| 15qE1 | 83002601 | 83002630 | *Cyb5r3* | None | n/a |
| 15qF2 | 100991141 | 100991170 | None | AGAGCGAGGC | High |
| 16qA1 | 4542011 | 4542170 | *Tcfap4* | GGACCGAGGC | High |
| 16qB1 | 25744591 | 25744670 | *Trp63* | TAAAGCAGGC | Low |
| 16qB5 | 51120081 | 51120260 | None | AGAACGAGGC | High |
| 16qC1.3 | 60152551 | 60152560 | *Epha6* | Too Short | n/a |
| 16qC1.3 | 63817641 | 63817760 | *Epha3* | GAAACGAGGT | Medium |
| 16qC3.3 | 81434881 | 81434960 | *Ncam2* | AGAAAAAGAG | Low |
| 16qC3.3 | 87293311 | 87293330 | None | Too Short | n/a |
| 16qC4 | 95562801 | 95562870 | *Erg* | AGAGGGGGAT | Low |
| 16qC4 | 96893341 | 96893440 | *Dscam* | AGACTGAGGC | High |
| 17qA1 | 11813721 | 11813740 | *Park2* | Too Short | n/a |
| 17qA3.3 | 26813341 | 26813590 | *Atp6v0e* | AGAGCGAGGC | High |
| 17qB1 | 34974028 | 34974070 | *Dom3z* | GAAGTGAGGC | High |
| 17qB1 | 39980121 | 39980370 | None | CAAGCGAGGA | Medium |
| 17qB1 | 39980500 | 39980870 | None | AAAGCCAGGC | Low |
| 17qB1 | 39981050 | 39981070 | None | Too short | n/a |
| 17qB1 | 39981390 | 39981790 | None | AGAGAGAGAC | Medium |
| 17qB1 | 39982060 | 39982180 | None | GGAAAGAGGC | Medium |
| 17qB1 | 39982470 | 39982590 | None | GAAGCCAGGA | Low |
| 17qB1 | 39982820 | 39982910 | None | TGAATGGTGC | Low |
| 17qB1 | 39983030 | 39983070 | None | TAAGAGAGGT | Medium |
| 17qB1 | 39983480 | 39983510 | None | GAAGGGGGTC | Low |
| 17qB1 | 39983730 | 39983810 | None | GGAGTGGGTA | Low |
| 17qB1 | 39984430 | 39984730 | None | GAATCGAGAA | Medium |
| 17qB1 | 39984830 | 39984930 | None | GGATCGGGGA | Low |
| 17qB1 | 39985050 | 39985680 | None | GGAGTGAGGT | Medium |
| 17qB3 | 45644131 | 45644150 | *Aars2* | Too Short | n/a |
| 17qD | 56579341 | 56579430 | *Ptprs* | ATGCTGAGGC | Low |
| 17qD | 57387951 | 57387980 | *Gpr108* | GGAAAGACGC | Low |
| 17qE3 | 80882401 | 80882420 | *Sos1* | Too Short | n/a |
| 18qA2 | 24178921 | 24179060 | *Zfp191* | AAAGCAGGGC | Low |
| 19qA | 3912981 | 3913030 | *Ndufs8* | AAACCGAGGT | Medium |
| 19qA | 10677451 | 10677540 | *Dak* | AGAGCGAGCC | Medium |
| 19qA | 12576481 | 12576510 | *Dtx4* | TGACAGAGAG | Medium |
| 19qA | 14445951 | 14446070 | None | AGAATGAGGC | High |
| 19qC3 | 41513581 | 41513680 | None | AAACCGAGGC | High |
| 19qD1 | 47800531 | 47800550 | *6330577E15Rik* | Too Short | n/a |
| XqA5 | 54646081 | 54646330 | *Rbmx* | GGAACGAGGC | High |
| XqE3 | 120777341 | 120777350 | None | Too Short | n/a |
| XqE3 | 120779291 | 120779310 | None | Too Short | n/a |
| XqE3 | 131543811 | 131543920 | *Zmat1* | AGAGCGAGGC | High |
| XqF3 | 148777341 | 148777510 | *Tspyl2* | ATAGCGAGGC | High |
| YqA1 | 2793071 | 2873170 | None | GGATGGAGGA | Medium |
| YqA1 | 2859620 | 2859630 | None | Too Short | n/a |
| YqA1 | 2876750 | 2876770 | None | Too Short | n/a |
